# Soybean fruit development and set at the node level under combined photoperiod and radiation conditions

**DOI:** 10.1093/jxb/erv475

**Published:** 2015-10-27

**Authors:** Magalí Nico, Anita I. Mantese, Daniel J. Miralles, Adriana G. Kantolic

**Affiliations:** ^1^Cátedra de Cultivos Industriales, Facultad de Agronomía, Universidad de Buenos Aires, Av. San Martín 4453, C1417DSE Buenos Aires, Argentina; ^2^Cátedra de Botánica General, Facultad de Agronomía, Universidad de Buenos Aires,Av. San Martín 4453, C1417DSE Buenos Aires, Argentina; ^3^Cátedra de Cerealicultura, Facultad de Agronomía, Universidad de Buenos Aires, Av. San Martín 4453, C1417DSE Buenos Aires, Argentina; ^4^CONICET (Consejo Nacional de Investigaciones Científicas y Técnicas), Argentina; ^5^IFEVA (Instituto de Investigaciones Fisiológicas y Ecológicas Vinculadas a la Agricultura), Av. San Martín 4453, C1417DSE Buenos Aires, Argentina

**Keywords:** Development, elongation, embryo, flowering, fructification, *Glycine max*, lag phase, node, photoperiod, pod set, radiation, seed filling, shade, soybean.

## Abstract

In soybean, long days during post-flowering increase seed number. This positive photoperiodic effect on seed number has been previously associated with increments in the amount of radiation accumulated during the crop cycle because long days extend the duration of the crop cycle. However, evidence of intra-nodal processes independent of the availability of assimilates suggests that photoperiodic effects at the node level might also contribute to pod set. This work aims to identify the main mechanisms responsible for the increase in pod number per node in response to long days; including the dynamics of flowering, pod development, growth and set at the node level. Long days increased pods per node on the main stems, by increasing pods on lateral racemes (usually dominated positions) at some main stem nodes. Long days lengthened the flowering period and thereby increased the number of opened flowers on lateral racemes. The flowering period was prolonged under long days because effective seed filling was delayed on primary racemes (dominant positions). Long days also delayed the development of flowers into pods with filling seeds, delaying the initiation of pod elongation without modifying pod elongation rate. The embryo development matched the external pod length irrespective of the pod’s chronological age. These results suggest that long days during post-flowering enhance pod number per node through a relief of the competition between pods of different hierarchy within the node. The photoperiodic effect on the development of dominant pods, delaying their elongation and therefore postponing their active growth, extends flowering and allows pod set at positions that are usually dominated.

## Highlight

Long days during post-flowering postpone elongation and active growth of dominant pods within a node, which extends flowering and allows pod set at usually dominated positions.

## Introduction

Soybean (*Glycine max* (L.) Merr.) pod number, which is an important yield component, is determined during a period that begins around flowering and extends through pod set and the beginning of the seed-filling period (Board and Tan, 1995; Jiang and Egli, 1995; Egli, 1997). The availability of assimilates during these post-flowering phases affects pod and seed number (Egli and Yu, 1991; Board *et al.*, 1995; Jiang and Egli, 1995; De Bruin and Pedersen, 2009). Thus, these post-flowering phases are often regarded as the critical period for yield determination (Egli, 1998).

Soybean is a short-day plant and both photoperiod and temperature control the duration of the whole crop cycle. Long photoperiods delay flowering (Borthwick and Parker, 1938; Hadley *et al.*, 1984; Upadhyay *et al.*, 1994; Zhang *et al.*, 2001) and soybean cultivars’ pre-flowering response to photoperiod is the base for their characterization into different maturity groups (Summerfield and Roberts, 1985). Long photoperiods also extend the duration of post-flowering phases (Thomas and Raper, 1976; Guiamet and Nakayama, 1984; Summerfield *et al.*, 1998; Han *et al.*, 2006; Kantolic and Slafer, 2007). The fact that soybean yield is mainly determined during post-flowering phases highlights the importance of the post-flowering photoperiodic response in the complex process of soybean yield determination.

Long days during post-flowering phases increase seed and pod number per square metre in soybean primarily due to an increase in the production of nodes and, secondly, due to increases in pods per node, without changing the number of seeds per pod (Guiamet and Nakayama, 1984; Morandi *et al.*, 1988; Kantolic and Slafer, 2001; Kantolic *et al.*, 2013; Nico *et al.*, 2015). Photoperiod extensions during post-flowering phases also increase the cumulative intercepted radiation and thereby enhance seed number (Kantolic and Slafer, 2005). In recent experiments, the effects of shading and photoperiod treatments on seeds per square metre could be explained through the differences in the cumulative intercepted radiation – when treatments were applied from the beginning of fructification onwards (Kantolic *et al.*, 2013). However, the spatial distribution of pods was not the same under shading and photoperiod treatments. When similar shading and photoperiod treatments were applied earlier, from flowering onwards, an additional direct photoperiodic effect (i.e. independent of the cumulative intercepted radiation) increased seed number (Nico *et al.*, 2015). This direct photoperiodic effect was related to the increased node production observed under long days.

At node level, pod and seed number exhibit a nonlinear saturation response to photosynthesis (Bruening and Egli, 1999, 2000) suggesting the existence of regulatory processes that are independent of the availability of assimilates. The survival of an individual pod is strongly influenced by the presence of other pods at the same node but is relatively independent of the presence and the photosynthesis of the subtending leaf (Egli, 2005) and the presence of pods at other nodes (Heitholt *et al.*, 1986). This evidence suggests that the interference among pods is an intra-nodal process, highlighting the importance of the spatial distribution of sinks at different nodes within the plant or canopy. Photoperiod alterations from the beginning of the reproductive period modify node production (Guiamet and Nakayama, 1984; Han *et al.*, 2006) and could thereby alter the spatial distribution of sinks among different nodes (Nico *et al.*, 2015).

Within a node, the first flowers that appear at basal positions of primary racemes are less likely to abort than those that appear later and/or at more distal or lateral positions (on secondary and tertiary racemes) (Brun and Betts, 1984; Heitholt *et al.*, 1986). Apparently the flowers that appear earlier produce hormones (such as indole-3-acetic acid) that induce abortion of flowers at distal positions, at least during their sensitive phase to abortion (Huff and Dybing, 1980). Fruits that reach their maximum length rarely abort (Heitholt *et al.*, 1986; Egli and Bruening, 2006a) and this usually occurs near the beginning of the linear phase of seed growth (Egli *et al.*, 1981; Egli, 1998). All this evidence demonstrates the important role of the temporal distribution of sinks within the node (Egli, 2005) and suggests that pod number could be enhanced by intrinsic or environmental factors that modify the temporal dynamics of pod production. One strong candidate to modify these dynamics is photoperiod, as some evidence showed that fruit elongation was anticipated when soybean plants were exposed to short photoperiods from flowering to maturity (Zheng *et al.*, 2003).

The evidence for photoperiodic effects on the temporal and spatial dynamics of soybean sinks suggests that photoperiod might affect pod set at the node level thus alleviating intra-nodal pod interactions. This work aims to identify the main mechanisms responsible for the increase in pods per node in response to long days including the dynamics of flowering, pod development, growth and set at the node level.

## Materials and methods

### Culture, experimental design and treatments

Research was conducted at the experimental field of the School of Agronomy, University of Buenos Aires (34°35′S, 58°29′W), during two growing seasons (2008/2009 and 2009/2010) using the commercial soybean cultivar NA 5009 RG (Nidera Argentina). This is an indeterminate cultivar of Maturity Group V, well adapted to and widely grown in the Rolling Pampas region of Argentina. Seeds were inoculated with *Bradyrhizobium* liquid inoculant and sown on two contrasting sowing dates: 25 January and 25 October 2009 in Exp1 and Exp2, respectively. Seedlings were thinned after emergence to a uniform density of 40 plants per square metre. Plots were irrigated using drip-line tubing to complement rainfall. Weeds, pests and diseases were chemically controlled following local agronomic practices. Each experiment was arranged in a randomized complete-block design with three replicates. Experimental plots consisted of six rows, 2.5 m long, with 0.35 m row spacing.

Treatments consisted of the factorial combinations of different shade and photoperiod levels applied from reproductive (R) stages (Fehr and Caviness, 1977) ‘beginning bloom’ (R1) through to ‘beginning maturity’ (R7). Before R1, all plants grew under natural photoperiod and radiation. Shaded treatments were achieved by installing commercial shade nets over the plots to reduce canopy photosynthesis (35% radiation reduction) without changing the spectral composition of light (red to far-red ratio of 1.2 underneath the shade, measured using a SKR 110 660/730 sensor, Skye Instruments Ltd). Unshaded control plots were maintained without the shade nets. Photoperiod treatments were achieved by exposing the plots to an artificially extended photoperiod, in relation to the natural photoperiod, by means of portable lighting structures that switched on and off automatically depending on the length of extension (Kantolic and Slafer, 2001). Plots were either kept under a natural photoperiod (control) or a photoperiod extended by 3h in relation to the natural photoperiod. Due to sowing date differences, the mean natural photoperiod in Exp2 was longer than in Exp1 (14.5h vs 12.4h, respectively); therefore, an intermediate photoperiod treatment extended by 1.5h in relation to the natural photoperiod was added in Exp2. This treatment was included to take into account that too long a photoperiod could saturate some photoperiodic responses (Kantolic and Slafer, 2005). More details of the environmental conditions during Exp1 and Exp2 are presented in Nico *et al.* (2015). Each lighting structure combined incandescent and fluorescent lamps that provided an extremely low photosynthetic photon flux density (400–700nm) of 4 μmol m^−2^ s^−1^ (measured on top of the canopy using a LI-COR Inc. quantum sensor) and a red to far-red ratio of 1.17 (measured using a SKR 110 660/730 sensor, Skye Instruments Ltd), which is similar to daylight (Holmes and Smith, 1977). Lighting structures and shade nets were always kept 20–30cm above the canopy.

### Measurements and estimated variables

**Fig. 1.**
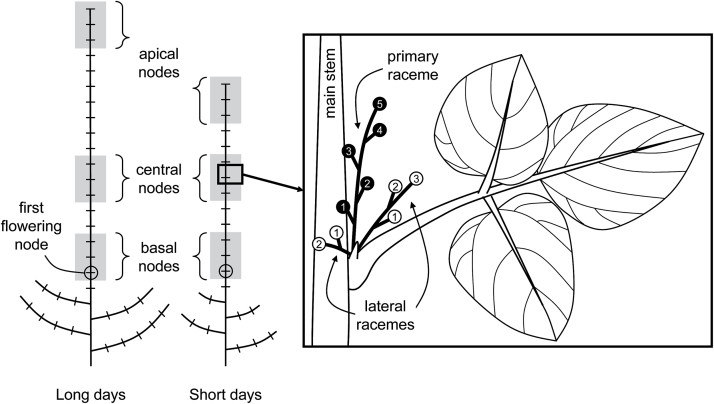
Schematic representation of soybean plants indicating the first flowering node (circled) and the basal, central and apical triplet of nodes observed on the main stems of plants under long and short days (left) and the localization of reproductive structures on primary and lateral racemes within the same node axil (right). Each number indicates the order of appearance within each raceme.

At R1, three plants were tagged within each plot. In each plant, measurements were made at three consecutive nodes located on a basal (starting where flowering began at R1), central (starting five nodes above the node where flowering began at R1) or apical section of the main stem (the three last nodes) (Fig. 1). The mean value of the three consecutive nodes within each position was used as a replicate. The apical section varied within experiments and treatments depending on the final number of nodes in the main stem.

Within each node, reproductive organs were counted three times per week on primary and lateral (secondary and tertiary) racemes separately (according to Torigoe *et al.*, 1982) (Fig. 1). In both experiments the reproductive organs were grouped into four categories according to their developmental stage: A, open flower; P_0_, pod <1cm long; P_1_, pod 1–2cm long; P_2_, pod 2–3cm long. In Exp2 four extra categories were included: P_3_, pod 3–4cm long; P_4_, pod 4–5cm long; P_5_, pod >5cm long; BF, pod with seeds >3mm long [considered the beginning of the effective seed filling, using a similar criterion to that used by Fehr and Caviness (1977) for the R5 stage defined at the plant level]. As flower abortion was negligible before the maximum number of open flowers was reached at each node, the total number of flowers was estimated from the maximum number of reproductive organs (flowers and pods) counted within each node. The final number of pods per node was counted at maturity. Pod set was estimated as the ratio between final pod number and total number of flowers.

The duration of flowering within each raceme was determined as the days between the first and last opened flower at each raceme. The pod lag phase of the first pod on the raceme was determined as the phase between the opening of the flower and the moment when the pod reached 2cm long. The first pod on primary racemes was considered the dominant pod at the node. For the first pod on primary racemes in Exp2, time to the beginning of seed filling was determined as the time between the opening of the flower and the moment when the seeds inside the pod reached 3mm.

All phase durations were corrected by temperature and expressed in thermal days (td) using the linear three-segmented function and cardinal temperatures proposed by Piper *et al.* (1996). Daily maximum and minimum temperatures were collected from a standard meteorological station located ~300 m away from the plots (Vantage Pro2, Davis Instruments, California, USA).

At full maturity [R8, as described by Fehr and Caviness (1977)], the three tagged plants within each plot were harvested. Pod and node number were counted on main stems and branches separately. Pods per node (PPN) were calculated as the ratio between total pods and total nodes per main stem or branch.

### Statistical analysis

A mixed linear model was fitted to all measured and estimated data using the *lme* procedure of the *nlme* package (Pinheiro *et al.*, 2012) of R v 3.1.3 (R Development Core Team, 2015) by restricted maximum likelihood estimation (REML). Factors ‘photoperiod’, ‘radiation’ and their interaction were included as fixed terms, while ‘experiment’, ‘block’ and ‘plant’ were considered as random terms. The factor ‘plant’ was nested in ‘block’ which was again nested in the factor ‘experiment’. Multiple comparisons between means were performed using a procedure equivalent to the LSD Fisher test with a significance level of α=0.05. The analysis was performed using InfoStat v2015 (Di Rienzo *et al.*, 2015), a user-friendly interface to the *nlme* package of R. Regression and path analysis were performed with InfoStat v2015 (Di Rienzo *et al.*, 2015).

### Light microscopy

In Exp2, flowers (A), small pods (P_1_ and P_2_) and transverse sections pods (P_3_, P_4_ and BF) were collected as they appeared on primary racemes of central nodes of unshaded plants under control or 3h extended photoperiod. Pods in the P_0_ category from plants under the extended photoperiod were collected successively every week until they reached the next category (P_1_).

Flowers and pods were cut with a clean double-sided razor blade and immediately fixed in formalin/acetic acid/ethanol for 48h. The fixed samples were dehydrated in an ethanol/xylol series and then infiltrated and embedded in paraffin (D’Ambrogio de Argüeso, 1986). The embedded material was sectioned transversally and serially (10–12 µm thick) using a Minot-type rotary microtome. The sections were stained with safranin and fast green in ethanol and mounted in Canada balsam. Sections were photographed with a Zeiss Axioplan optical microscope (Oberkochen, Germany) and analysed with the Zeiss AxioCam ERc 5s software (Jena, Germany). Three samples from each reproductive organ category were collected per plot but, unless otherwise necessary, only one sample per plot was analysed.

## Results

### Nodes, flowers and pods

Photoperiod extension during post-flowering delayed reproductive development and extended the crop’s cycle; therefore, nodes continued appearing for a longer time under the extended photoperiod (data not shown). As a result, plants under an extended photoperiod had 3.4–5.8 extra nodes on their main stems, depending on the magnitude of photoperiod extension (Table 1). In addition, plants under an extended photoperiod had 0.22–0.34 extra pods per main stem-node, resulting (together with the extra nodes) in 12 extra pods per plant allocated on the main stems.

**Table 1.** Means of node number per plant, pods per plant and pods per node (PPN) on main stems or branches. Minor and main effects of the factorial combination of photoperiod and radiation treatments and *P*-value of the estimated fixed effects are given

	Main stem						Branches					
	Nodes		Pods		PPN		Nodes		Pods		PPN	
Unshaded												
Control	18 .0	d	25 .0	b	1 .35	ab	20 .8	bc	22 .7	ab	1 .08	a
1.5 h	20 .2	c	34 .6	a	1 .63	a	18 .6	bc	14 .6	bc	0 .81	ab
3 h	23 .2	ab	37 .0	a	1 .54	a	30 .8	a	32 .3	a	0 .91	ab
Shaded												
Control	17 .4	d	20 .1	b	1 .15	b	14 .6	c	10 .8	c	0 .81	b
1.5 h	22 .0	bc	35 .3	a	1 .56	a	26 .1	ab	21 .0	abc	0 .84	ab
3 h	23 .9	a	33 .8	a	1 .39	ab	19 .6	bc	20 .1	bc	0 .91	ab
												
Control	17 .7	C	22 .6	B	1 .25	B	17 .7	B	16 .7	B	0 .95	A
1.5 h	21 .1	B	35 .0	A	1 .59	A	22 .4	AB	17 .8	AB	0 .83	A
3 h	23 .5	A	35 .4	A	1 .47	A	25 .2	A	26 .2	A	0 .91	A
												
Unshaded	20 .4	A	32 .2	A	1 .51	A	23 .4	A	23 .2	A	0 .94	A
Shaded	21 .1	A	29 .7	A	1 .37	A	20 .1	A	17 .3	A	0 .85	A
Intercept	***		***		***		**		*		***	
Photoperiod	***		***		***		*		*		ns	
Radiation	ns		ns		ns		ns		ns		ns	
Photoperiod×radiation	ns		ns		ns		**		ns		ns	

ns, not significant; *, *P*<0.1; **, *P*<0.05; ***, *P*<0.01. Different letters within a column indicate significant differences (*P*<0.05) according to LSD Fisher multiple comparison test; lower case differentiates the minor effects of factorial combination of photoperiod and radiation treatments and upper case differentiates the main effects of photoperiod or shading separately.

Branch-nodes per plant were also higher under an extended photoperiod, but there was an interaction between photoperiod and radiation. In comparison with the natural photoperiod, branch-nodes per plant were significantly increased by the 1.5h photoperiod extension only in the shaded treatments and by the 3h extension only in the unshaded treatments (Table 1). Photoperiod extension had no significant effect on the number of pods per branch-node, but it tended to be lower under the 1.5h treatment. As a result, the 3h photoperiod extension had 9.5 more pods on branches than the control (through its effect on branch-nodes), while the 1.5h extension had no significant effects on the final number of pods on branches.

Shading had no effect on plant development and main stem nodes per plant, but tended to reduce the number of branch-nodes per plant (*P*=0.26) (Table 1). The number of pods per node also tended to be slightly lower in shaded plants (*P*=0.11 on main stems and *P*=0.34 on branches). The mean number of pods per plant on main stems and branches of shaded plants was always lower compared to the plants grown under full radiation, but differences were not significant (*P*=0.30 on main stems and *P*=0.12 on branches).

The number of opened flowers per node on primary racemes was relatively stable and ranged from 1.8 to 3.3 flowers per raceme within node positions and photoperiod treatments (Table 2). The range of opened flowers on primary racemes was higher at the apical nodes, in comparison with other node positions. At this position of the plant, photoperiod extension increased flower production. These extra flowers produced on primary racemes at the apical nodes resulted in 0.3 and 0.5 extra pods under the 1.5 and 3h photoperiod extension, respectively. In contrast, at the basal and central nodes, photoperiod extension did not affect flower opening at the apical nodes. There was an interaction between photoperiod and shading on pod number determination on the primary racemes at the central and basal nodes. At the central nodes, pod number on the primary racemes was reduced (0.8 less pods) only in the unshaded 3h photoperiod extension treatment. At the basal nodes, the 3h photoperiod extension reduced the number of pods on the primary racemes under both radiation levels, while the 1.5h photoperiod extension only reduced it under shade (~0.7 less pods). Shading had an additional detrimental effect on pod number on the primary racemes of basal nodes (0.4 less pods).

**Table 2.** Means of opened flower number and final number of pods per node on primary or lateral racemes at basal, central or apical nodes of the main stem. Minor and main effects of the factorial combination of photoperiod and radiation treatments and *P*-value of the estimated fixed effects are given

	Basal nodes								Central nodes								Apical nodes							
	Primary racemes				Lateral racemes				Primary racemes				Lateral racemes				Primary racemes				Lateral racemes			
	Flowers		Pods		Flowers		Pods		Flowers		Pods		Flowers		Pods		Flowers		Pods		Flowers		Pods	
Unshaded																								
Control	3 .1	a	1 .6	a	4 .2	b	1 .1	ab	2 .7	a	2 .0	a	1 .4	b	0 .6	bc	1 .8	b	0 .6	cd	0 .5	a	0 .1	ab
1.5 h	2 .9	a	1 .3	ab	7 .0	a	0 .6	b	2 .4	b	1 .7	ab	2 .5	a	1 .3	ab	2 .3	b	0 .9	bcd	0 .7	a	0 .2	a
3 h	3 .2	a	0 .9	c	6 .6	a	1 .9	a	2 .7	ab	1 .2	b	2 .6	a	1 .1	ab	3 .4	a	1 .2	a	0 .8	a	0 .1	ab
Shaded																								
Control	3 .1	a	1 .3	b	2 .7	b	0 .6	b	2 .7	ab	1 .5	b	1 .1	b	0 .3	c	1 .8	b	0 .5	d	0 .4	a	0 .1	ab
1.5 h	2 .8	a	0 .6	d	5 .0	ab	1 .1	ab	2 .6	ab	1 .7	ab	3 .0	a	1 .6	a	2 .3	b	0 .9	abc	0 .3	a	0 .0	ab
3 h	2 .8	a	0 .5	d	4 .5	ab	1 .8	a	2 .6	ab	1 .5	b	2 .3	a	1 .3	ab	3 .2	a	1 .0	ab	0 .8	a	0 .0	b
																								
Control	3 .1	A	1 .5	A	3 .5	B	0 .9	B	2 .7	A	1 .7	A	1 .3	B	0 .5	B	1 .8	B	0 .6	B	0 .4	B	0 .1	A
1.5 h	2 .9	A	0 .9	B	6 .0	A	0 .9	B	2 .5	A	1 .7	A	2 .8	A	1 .5	A	2 .3	B	0 .9	A	0 .5	AB	0 .1	A
3 h	3 .0	A	0 .7	C	5 .6	A	1 .9	A	2 .6	A	1 .4	B	2 .4	A	1 .2	A	3 .3	A	1 .1	A	0 .8	A	0 .1	A
																								
Unshaded	3 .0	A	1 .3	A	6 .0	A	1 .2	A	2 .6	A	1 .6	A	2 .1	A	1 .0	A	2 .5	A	0 .9	A	0 .7	A	0 .1	A
Shaded	2 .9	A	0 .8	B	4 .1	B	1 .2	A	2 .6	A	1 .6	A	2 .2	A	1 .0	A	2 .5	A	0 .8	A	0 .5	A	0 .0	B
Intercept	***		***		**		ns		***		***		***		**		***		***		***		**	
Photoperiod	ns		***		***		***		ns		**		***		***		***		***		*		ns	
Radiation	ns		***		***		ns		ns		ns		ns		ns		ns		ns		ns		**	
Photoperiod×radiation	ns		*		ns		ns		ns		*		ns		ns		ns		ns		ns		ns	

ns, not significant; *, *P*<0.1; **, *P*<0.05; ***, *P*<0.01. Different letters within a column indicate significant differences (*P*<0.05) according to LSD Fisher multiple comparison test; lower case differentiates the minor effects of factorial combination of photoperiod and radiation treatments and upper case differentiates the main effects of photoperiod or shading separately.

In contrast to that observed on the primary racemes, the number of opened flowers on the lateral racemes showed considerable variation, ranging from 0.3 to 7.0 flowers per raceme depending on the node position and the treatment (Table 2). The number of opened flowers on lateral racemes was high at the basal nodes and almost null at the apical nodes. Shading reduced the number of opened flowers on the lateral racemes only at the basal nodes. Photoperiod extension increased opened flowers at all node positions (0.1–2.5 extra flowers per raceme). However, the magnitude of the photoperiodic effect was higher at the basal and central nodes than at the apical nodes, which had consistently fewer flowers on the lateral racemes (0.3–0.8 flowers per raceme). At the apical nodes, pod number on the lateral racemes was not modified by the photoperiod treatments and was reduced by shading; actually, pods rarely set on the lateral racemes of the apical nodes under any treatment (0–0.2 pods per raceme). At the central nodes, the extra flowers opened on the lateral racemes resulted in 0.7–1.0 extra pods under both photoperiod extension treatments. At the basal nodes, pod number on the lateral racemes was only increased by the 3h extension treatment.

Summarizing, the photoperiod extension had a positive effect on pod number on the lateral racemes and, simultaneously, a negative effect on primary racemes. These opposite effects had an overall positive effect on the plant, with an average increase of 0.32 pods per node. The variation in the response of pods per node to photoperiod and shading at different racemes and node positions within the plant (Table 2) explains the mild but significant effects observed at the plant level (Table 1).

### Dynamics of flowering and pod development at the node level

As treatments were imposed immediately after the beginning of flowering (R1), flower opening at successive upper nodes advanced alongside under both photoperiodic and shading treatments. Therefore, flowering also began simultaneously on the primary racemes of central nodes, located three nodes above the basal ones. As plants had a different number of nodes on their main stems depending on the photoperiod treatment (Table 1), flowering and pod setting at the apical nodes occurred at different moments and under different environmental conditions. In Exp1, apical nodes flowered on 16 March 2009 (summer ending) under control photoperiod and 11 days (6 td) later under 3h extended photoperiod. In Exp2, apical nodes flowered on 11 January 2010 (summer beginning) under the control photoperiod and 14 and 26 days (14 and 25 td) later under the 1.5 and 3h extended photoperiod, respectively. As expected, flowering on lateral racemes within each nodal position began later than the flowering on the primary raceme.

**Fig. 2.**
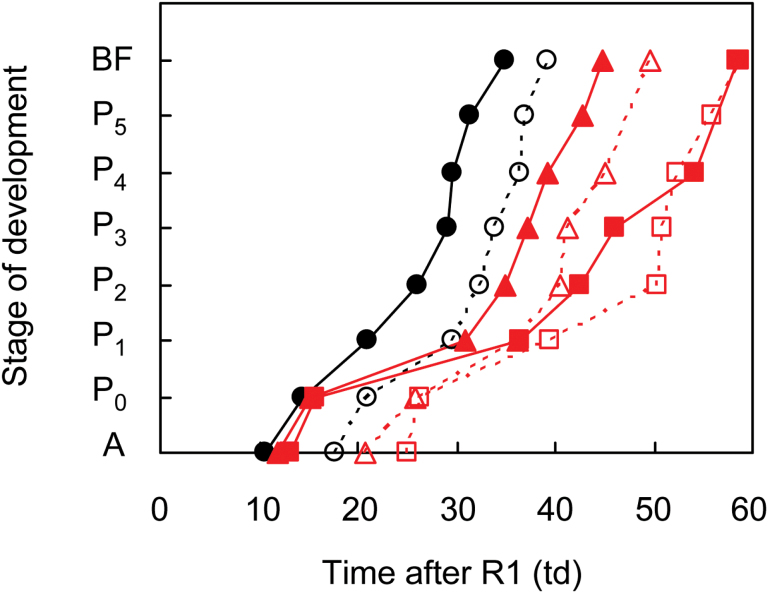
Developmental stage of the first fruit at central nodes of main stems as a function of thermal days (td) after flowering (R1) for primary (filled symbols and line) and lateral racemes (empty symbols and dotted line) of unshaded plants grown under control (circles), 1.5h (triangles) or 3h extended photoperiod (squares) in Exp2. For other node positions on the main stem, shaded plants and Exp1 see Supplementary Fig. S1. Developmental scale: A, open flower; P_0,_ pod <1cm long; P_1,_ pod 1–2cm long; P_2,_ pod 2–3cm long; P_3,_ pod 3–4cm long; P_4,_ pod 4–5cm long; P_5,_ pod >5cm long; BF, pod with seeds >3mm long. (This figure is available in colour at *JXB* online.)

Post-flowering photoperiod extension delayed individual fruit development from opened flower stage to the beginning of seed filling [Fig. 2 and Supplementary Fig. S1 (available at *JXB* online)]. Within all the developmental stages studied, the phase between P_0_ and P_1_ was the most responsive one to photoperiod extension. The developmental rate from P_1_ onwards was relatively stable for primary and lateral racemes for all photoperiod and shading treatments. Therefore, the pod lag phase seems responsible for the differences observed in time to beginning of seed filling (BF) between simultaneous-flowering primary racemes of the control and extended photoperiod treatments.

On the primary and lateral racemes of basal and central node positions photoperiod extension significantly increased the duration of the pod lag phase from 3 to 23 td depending on the photoperiod treatment and the node position within the plant (Table 3). The same tendency was observed at the apical nodes, but it was not statistically significant. The prolongation of the pod lag phase, in response to photoperiod extension, was stronger at the basal nodes compared to the central ones and on the primary racemes compared to the lateral ones. Shading also increased the pod lag phase at the basal and central nodes, but only on the primary racemes.

**Table 3.** Mean pod lag phase duration of the first fruit on primary and lateral racemes at basal, central or apical nodes of the main stem of plants. Minor and main effects of the factorial combination of photoperiod and radiation treatments and *P*-value of the estimated fixed effects are given

	Basal nodes				Central nodes				Apical nodes^†^	
	Primary racemes		Lateral racemes		Primary racemes		Lateral racemes		Primary racemes	
Unshaded										
Control	19 .09	d	8 .85	b	10 .63	d	8 .62	c	10 .48	a
1.5 h	30 .16	c	19 .10	a	20 .32	bc	14 .12	bc	12 .27	a
3 h	38 .35	b	25 .64	a	26 .17	ab	21 .34	a	14 .43	a
Shaded										
Control	19 .94	d	8 .29	b	12 .65	cd	14 .34	bc	9 .53	a
1.5 h	40 .48	ab	25 .06	a	27 .83	ab	15 .07	bc	14 .45	a
3 h	46 .84	a	25 .72	a	29 .19	a	18 .40	ab	12 .45	a
										
Control	19 .52	C	8 .57	B	11 .64	B	11 .48	B	10 .01	A
1.5 h	35 .32	B	22 .08	A	24 .07	A	14 .59	B	13 .36	A
3 h	42 .60	A	25 .68	A	27 .68	A	19 .87	A	13 .44	A
										
Unshaded	29 .20	B	17 .86	A	19 .04	A	14 .70	A	12 .39	A
Shaded	35 .75	A	19 .69	A	23 .23	A	15 .93	A	12 .14	A
Intercept	**		**		**		**		***	
Photoperiod	***		***		***		***		ns	
Radiation	***		ns		*		ns		ns	
Photoperiod×radiation	ns		ns		ns		ns		ns	

† The model could not be estimated for the lateral racemes at apical nodes because few pods developed at that position.

ns, not significant; *, *P*<0.1; **, *P*<0.05; ***, *P*<0.01. Different letters within a column indicate significant differences (*P*<0.05) according to LSD Fisher multiple comparison test; lower case differentiates the minor effects of factorial combination of photoperiod and radiation treatments and upper case differentiates the main effects of photoperiod or shading separately.

**Fig. 3.**
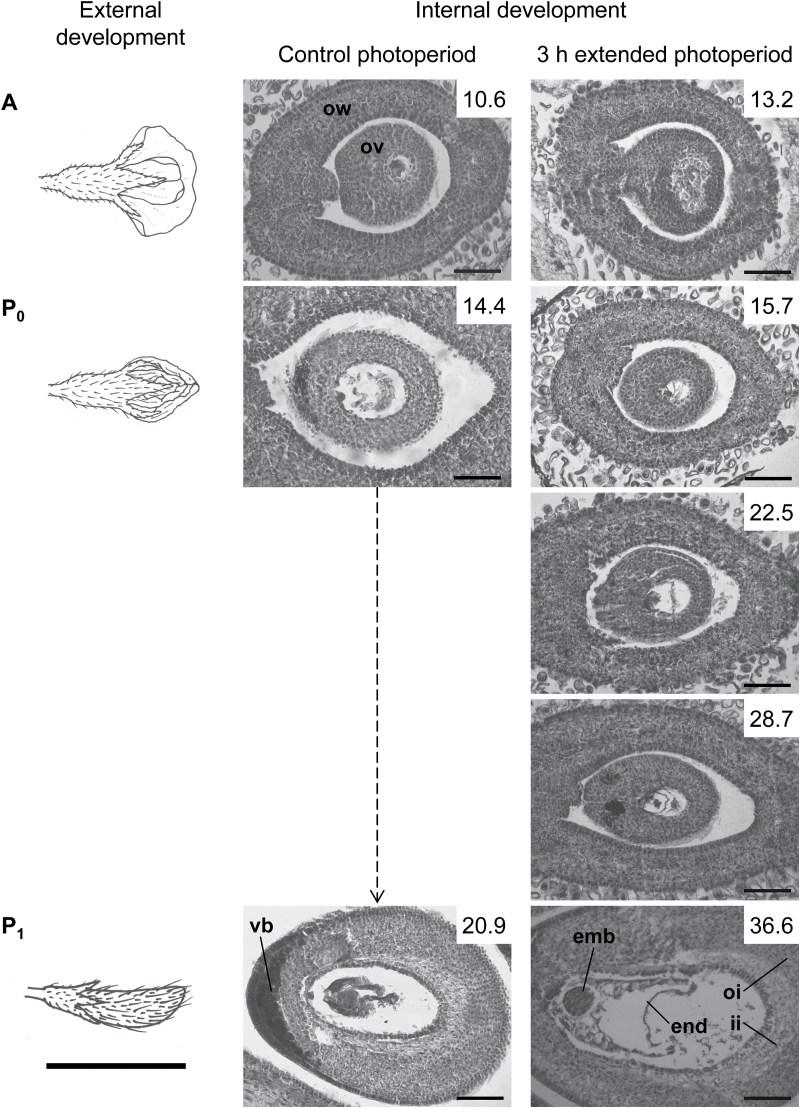

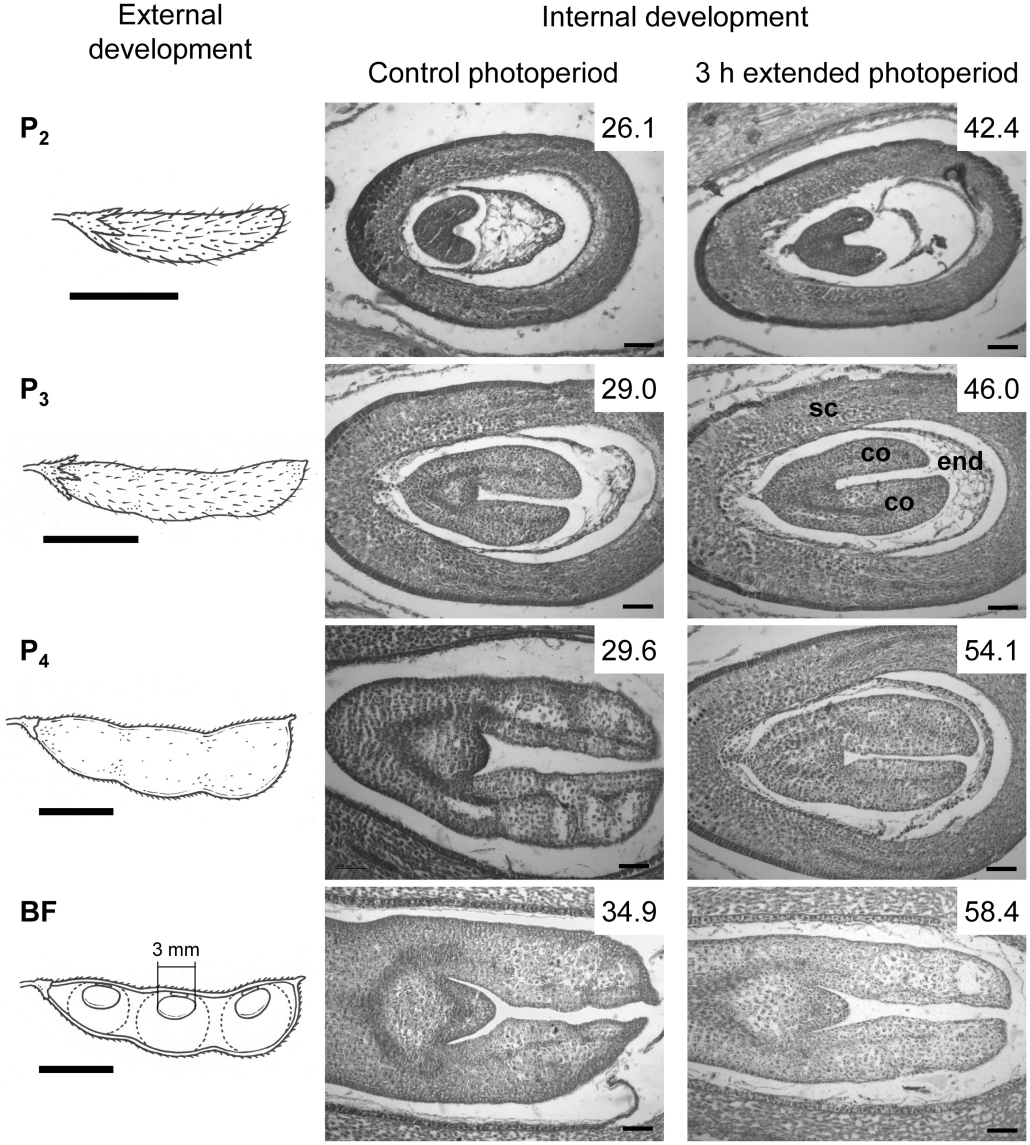
External and internal development of the first reproductive organ on the primary raceme at a central main stem node of unshaded plants under control or 3h extended photoperiod in Exp2. Cross sections of reproductive organs at: A, flower opening; P_0,_ pod <1cm long; P_1,_ pod 1–2cm long; P_2,_ pod 2–3cm long; P_3,_ pod 3–4cm long; P_4,_ pod 4–5cm long; BF, pod with seeds >3mm long. Pods in the P_0_ category from plants under extended photoperiod were collected successively every week until they reached the next category (P_1_). Boxed numbers are the days after R1 when each developmental state was reached. External development bar, 1cm. Internal development bar, 100 µm. ow, ovary wall; ov, ovary; vb, vascular bundles; emb, embryo; end, endosperm; oi, outer integument; ii, inner integument; sc, seed coat; co, cotyledon.

Successive microscopic observations during the pod lag phase and pod elongation revealed a similar embryo and pod wall development at the same external stage (A, P_0_, P_1_, P_2_, P_3_, P_4_ and BF), regardless of the pod’s chronological age (Fig. 3). The embryo reached the ‘globe’ stage [as pictured by Carlson and Lersten (2004)] when the pod’s length was 1cm, irrespective of the time elapsed from its flowering. When pods were 2cm long, embryos were at the ‘heart’ stage [as pictured by Carlson and Lersten (2004)] under natural and extended photoperiods, irrespective of the duration of the pod’s lag phase. These results indicate that both pod and embryo developments were delayed under extended photoperiods and that the internal ovule and embryo development correlate well with the length of the pod or seed.

### Relationship between pod development and pod number

**Fig. 4.**
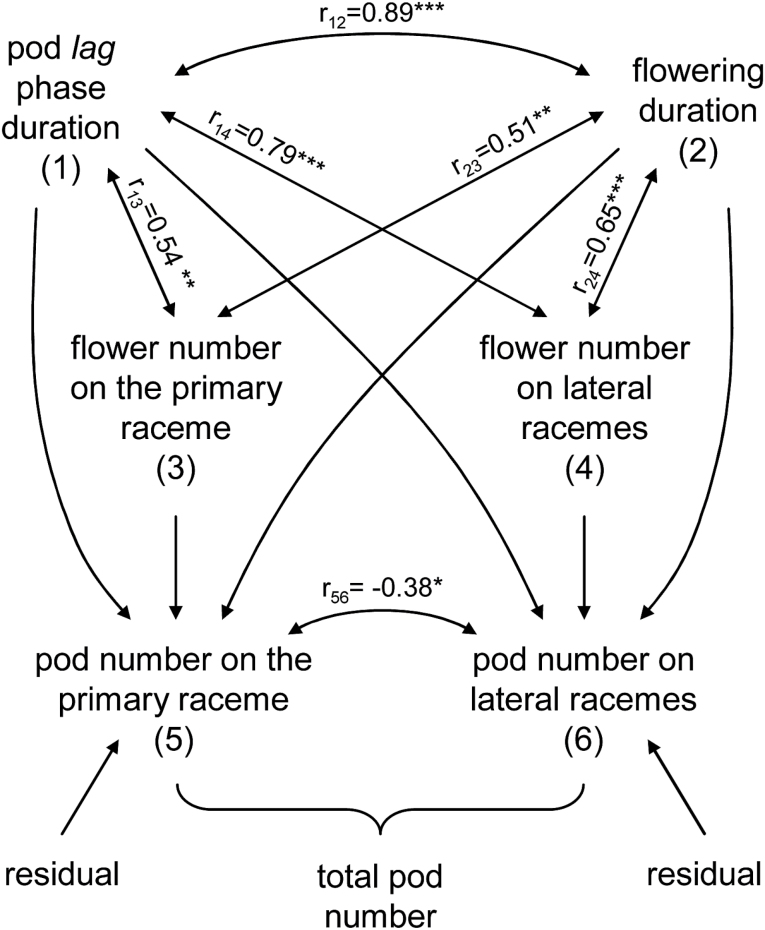
Two path diagrams showing causal relationships between: pod number on primary (5) and lateral racemes (6) and the component variables: pod lag phase duration (1), flowering duration (2) and flower number on primary (3) and lateral racemes (4). All variables are expressed on node basis and include basal, central and apical node data from both experiments. The double-arrowed lines indicate mutual association as measured by correlation coefficients (r) between two variables (subscripts) and the single-arrowed lines represent direct influence as measured by path coefficients. Additionally, the correlation between both response variables is shown. r-value significant at the *0.05, **0.01 or ***0.001 probability levels (*n*=30).

Photoperiod extension increased pod number at usually dominated positions within the node (lateral racemes) and delayed pod elongation at dominant positions (the primary raceme). To test the association between these two processes we analysed all the data using two-path analysis, one for the pods on primary racemes and another for pods on lateral racemes, including flowering duration and flower number data (Fig. 4).

**Fig. 5.**
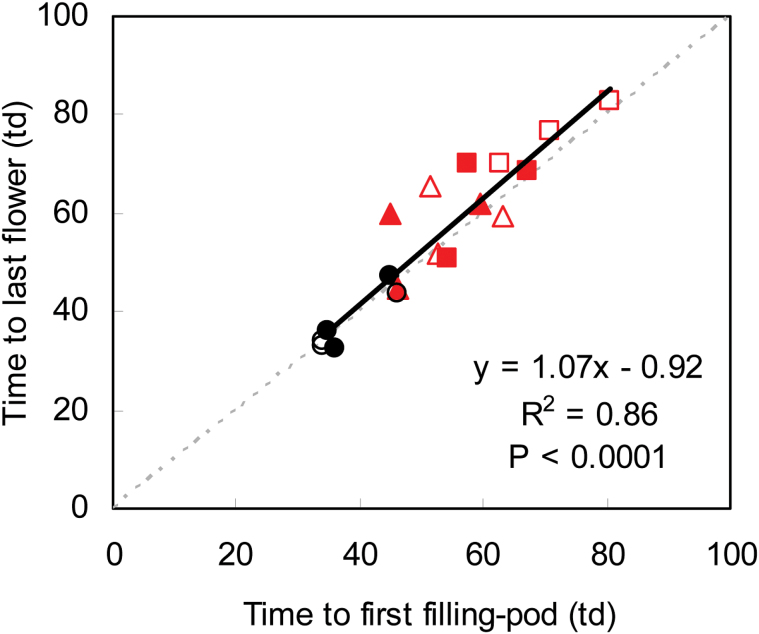
Relationship between time to last flower and time to first filling pod (in thermal days, td) at basal, central and apical nodes of the main stem of plants under full radiation (filled symbols) or shade (empty symbols) and control (circles), 1.5h (triangles) or 3h extended photoperiod (squares) in Exp2. (This figure is available in colour at *JXB* online.)

The duration of the pod lag phase and flowering were correlated to each other and were also correlated with the number of opened flowers on each raceme (Fig. 4). Surprisingly, the correlation in the number of flowers with the duration of the pod lag phase was higher than its correlation with the duration of the flowering period. In fact, within each node, flowering stopped when seed filling began (data only available for Exp2) as the fitted relationship between these dates (Fig. 5) was not significantly different from the identity line (95% confidence interval: 0.84–1.30).

No significant correlation was found between flower and pod number on the primary raceme (Table 4). However, pod number was negatively correlated with the duration of the flowering period and the pod lag phase. These correlations were low but significant and were caused by the strong direct negative effect of the duration of the pod lag phase on pod set on primary racemes (Supplementary Fig. S2).

**Table 4.** Direct and indirect path coefficients of pod lag phase and flowering duration (thermal days) and flower number on pod number on primary or lateral racemes. Correlation and *P*-value of the correlation between the three variables associated with the dynamics of pod setting and pod number are given

Trait	Direct effect of trait on pod number	Indirect effect of trait on pod number			Correlation (r) with pod number	*P*-value
		Pod lag phase duration	Flowering duration	Flower number		
**Primary raceme**						
Pod lag phase duration	−0.75	-	0.08	0.09	−0.59	0.0007
Flowering duration	0.09	−0.67	-	0.08	−0.50	0.0048
Flower number	0.16	−0.40	0.04	-	−0.20	0.2940
**Lateral racemes**						
Pod lag phase duration	0.17	-	0.32	0.39	0.88	<0.0001
Flowering duration	0.36	0.15	-	0.32	0.83	<0.0001
Flower number	0.49	0.13	0.23	-	0.86	<0.0001

Otherwise, on the lateral racemes high and significant correlations were found between the three component variables (pod lag phase, flowering duration and flower number) and pod number (Table 4). The number of flowers had a high correlation with pod number on lateral racemes that was mainly due to its direct effect. Flowering duration had a high correlation with pod number on lateral racemes due to its own direct effect and indirect effects (mediated by the number of flowers). The duration of the pod lag phase had a higher correlation with the number of pods on lateral racemes, mainly through its indirect effects through flowering duration and the number of flowers (Supplementary Fig. S2).

Given the negative effect of the duration of the pod lag phase on the number of pods on primary racemes and the inverse positive effect on the number of pods on lateral racemes, a negative correlation is expected between the number of pods on primary and lateral racemes; however, this correlation was low. The total number of pods per node was highly correlated with the number of pods on the lateral raceme (r=0.86, *P*<0.01) and poorly correlated with the number of pods on the primary raceme (r=0.14, *P*=0.45). Even though more pods were usually located on the primary than on the lateral racemes within a node (67% vs 33%, respectively), mean pod number was less affected by treatments on the primary racemes compared with the lateral racemes (range explored: 0.4–2.0 vs 0.0–3.2). Photoperiod affected pod number on lateral racemes through its effects on individual pod development (pod lag phase duration).

## Discussion

Our study revealed that photoperiod extension during post-flowering increased the number of pods per node, mainly by increasing pod number on the lateral racemes at some main stem nodes. Pod number on the lateral racemes was increased when photoperiod was extended because (i) more flowers opened and (ii) more pods set on those racemes. Photoperiod extension also delayed individual pod elongation and the beginning of seed filling, which started once the pods reached their maximum size. More flowers opened on the lateral racemes, due to the extension of the flowering period associated with the delay in the effective seed filling period at that node (on the primary racemes). These associations and possible photoperiodic effects on pod development that might increase pod number at the node level constitute a novel finding that is supported by many results of the present work.

In our experiments, reductions in incident radiation from flowering onwards only depressed flower production and pod setting at the basal nodes and these negative effects were diluted at the plant level. Previously, Jiang and Egli (1993) also observed that shading treatments of 30% of reduced incident radiation did not reduce flower number per node and pods per plant, while shading of 63% of reduced incident radiation reduced pods per plants as a result of fewer flowers per node and more flower and pod abscission.

As expected, plants under an extended photoperiod had more pods per node on their main stems, as previously reported (Guiamet and Nakayama, 1984; Morandi *et al.*, 1988; Kantolic and Slafer, 2001). This effect was observed in both experiments (which were sown in different dates) even though the environmental conditions and the number of nodes and pods per m^2^ were different between experiments (Nico *et al.*, 2015). A more detailed analysis at the node level at different positions of the main stem revealed that the magnitude and significance of the photoperiodic effect was variable between main-stem node positions, as recently reported by Kantolic *et al.* (2013). Additionally, we found a clear differential effect of long days on primary and lateral racemes that, to our knowledge, has not been previously reported. At some node positions (the earliest flowering ones), photoperiod extension reduced pod number on the primary racemes but this negative photoperiodic effect was usually compensated by a positive effect on pod production on lateral racemes. The negative effect on these primary racemes could be related to their extremely long pod lag phase (flowers took from 1 to 2 months to begin their fructification) in which flowers or small pods, which are still susceptible to abortion, could have been exposed to the environment’s adversities. These findings suggest the existence of a compromise between pod set at dominant and dominated positions to maximize pod production at the node level.

When exposed to long days after flowering, some soybean varieties have shown flowering reversion (Han *et al.*, 1998; Washburn and Thomas, 2000; Wu *et al.*, 2006; Jiang *et al.*, 2011). However, no evidence of this phenomenon was observed in the present work, so photoperiodic effects on pod development were apparently not linked to flowering reversion.

In our experiments, the photoperiodic effect on pod number on lateral racemes was associated with increases in both the number of opened flowers and pod set. Van Schaik and Probst (1958) also found that long days increased the number of flowers per node but, in contrast to our work, pod set depended on the magnitude of the photoperiod extension (and also the temperature): when photoperiods were too long or temperature was too high, the negative effect of flower and pod shedding cancelled the positive effect of enhanced flower production.

The number of opened flowers on primary racemes presented low variation as observed in the number of pods. In the present study, photoperiod extension treatments were imposed after R1, when flower differentiation culminates on primary racemes (but continues on lateral racemes) (Saitoh *et al*., 1998, 1999). Thereby, we may not have observed any effect of photoperiod extension on the number opened flowers on primary racemes if this response was associated with the differentiation of flower primordia. Besides the aforementioned effect of photoperiod, Egli and Bruening (2002a) also observed that at isolated nodes the number of flowers on the primary raceme seemed fixed at a relatively modest number, implying a relatively short flowering period, while the lateral racemes had a great potential to increase the length of the period and thereby to produce a large number of flowers per node. Thus, the extension of the flowering period and the enhancement of flower number at the node level, seem to depend on the lateral racemes. However, usually only a small proportion of the sub-racemes’ potential is utilized (Gai *et al.*, 1984; Jiang and Egli, 1993) perhaps because flowers on lateral-racemes are ‘weak’ sinks, commonly dominated by earlier and larger pods on the primary raceme.

At the whole plant level, a positive linear relationship between flowering duration and the number of flowers has been found when photoperiod was manipulated (van Schaik and Probst, 1958; Summerfield *et al.*, 1998). Under a natural photoperiod, Dybing (1994) found that the total number of flowers was more related to the flowering rate than to its duration. At node level, we confirmed the positive relationship between flowering duration and the number of opened flowers, revealing that plants under long photoperiods have long flowering periods not just because they have more flowering nodes, but also because flowering lasts longer at each node. We found that the flowering period was extended due to the appearance of flowers on lateral racemes. Unfortunately, Dybing (1994) – who found a weak relationship between flowering duration and the number of flowers – did not count the number of flowers on lateral racemes.

At each node, the flowering period was prolonged in accord with the delay of pod development under long photoperiods. The last flower at any node studied (located on the lateral raceme) opened at the same day as effective seed filling started at that node (when seeds were >3mm). Egli and Bruening (2002b) also observed this association between flowering end and the beginning of the linear phase of seed growth at phloem-isolated soybean nodes. This correspondence was attributed to a competition between sinks because, when seeds enter into the linear phase of growth and accumulate assimilates at maximum rate, they become a relatively large reproductive sink that may limit flowering (Spaeth and Sinclair, 1984).

Hierarchies established at node level cause the inhibition of late-appearing flowers or small pods by earlier and/or larger pods (Huff and Dybing, 1980; Brun and Betts, 1984; Heitholt *et al.*, 1986; Egli and Bruening, 2002b, 2006a, *b*). The simultaneous growth of pods of different hierarchies (position and age) has been postulated as a critical aspect of assimilate utilization (Egli and Bruening, 2002a). Even though flowers are normally produced in excess, the dynamics of flower production have been proposed as an important aspect in the complex process of pod and seed number determination. By delaying pod development, long days could be alleviating, or at least postponing, the interaction between dominant and dominated pods. Photoperiod effects on pod elongation and node appearance might have modified the temporal dynamics of the source-sink ratio at the node level. However, Nico *et al.* (2015) found that, at the full seed stage [R6, as described by Fehr and Caviness (1977)], the source-sink ratio at the crop level was not affected by photoperiod.

Furthermore, the inhibition of late-appearing flowers or small pods by earlier or larger pods has been largely studied, and several mechanisms and putative signals have been proposed. Huff and Dybing (1980), propose that flower abortion could be caused by hormonal induction suggesting indole-3-acetic acid as the candidate hormone. Abscisic acid could also be involved, because it has an inhibitory role on flowering (Bernier *et al.*, 1993) and the concentration of abscisic acid in seeds has been found to increase more slowly and peak later with night interruption treatments (Morandi *et al.*, 1990).

Even though the interaction between dominant and dominated pods is evident, it is still not clear whether there is an optimal temporal flowering profile in soybean (Egli, 2005) as both long (Egli and Bruening, 2000; Kantolic and Slafer, 2001) and short flowering periods (Egli and Bruening, 2002a) have been associated with increased pod set. The rapid increase in assimilate utilization by older pods make them the preferred sink and causes the abortion of late flowers at distal positions. Therefore, Egli and Bruening (2002a) proposed a strategy to increase pods per node synchronizing the production of many early flowers that would grow together rapidly. However, in the experiments by Egli and Bruening (2002a) ‘synchronous flowers’ were located at three different nodes fed by a single leaf at an isolated node system. Therefore, these synchronous flowers were not only temporally uncoupled from pods in active growth but also spatially detached. Our results suggest that there is another possible strategy to increase pod number per node based on the idea proposed by Egli and Bruening (2002a). Instead of looking for more synchronous and earlier flowering at the dominated positions, we propose to obtain synchronous development delaying the growth of the dominant pods. Thus, more flowers will be already opened at the time of rapid pod and seed growth, and they would also grow together, although not rapidly. In fact, recent modelling with SOYPODP [a whole plant model that assembles SOYPOD node units by Egli (2015)] revealed that lengthening the sensitive period of pod growth (pod lag phase) diminishes the competition for assimilates between pods of different age, increasing pod set and the number of pods per plant. We suggest, based on our studies, that the delay in pod development when plants are exposed to long days increases the potential number of seeds through the avoidance of competition for assimilates or signals triggered during the beginning of active pod and seed growth.

Post-flowering photoperiod extension effects were not alike during all pod developmental phases. We mentioned before that long days delayed seed filling, which begins once pods have reached their final length and width. Pod elongation does not begin immediately after flowering because there is a ‘pod lag phase’ that is considerably longer in soybean compared with other legumes (Zheng *et al.*, 2003). Photoperiod extension delayed the onset of pod elongation, prolonging the pod lag phase.

Under the natural photoperiod, duration of the pod lag phase differed according to the pod’s position on the plant (basal>central>apical nodes, primary>lateral racemes), being longer at the earliest flowering positions of the plant. Zheng *et al*. (2003, 2004) also found that the pod lag phase was shorter on lateral than primary racemes. The photoperiodic prolongation of the pod lag phase was greater on those pods which also had longer pod lag phases under natural photoperiod according to their node position and raceme. As the natural photoperiod diminished when the crop season advanced (and therefore the extended photoperiod did so as well), photoperiod was shorter when the later flowers opened. We found that the duration of a pod’s lag phase was related to the photoperiod explored when the flower was opened but its position on the main stem was also important, as late flowering positions were less sensitive and/or had less plasticity to respond to photoperiod (Supplementary Fig. S3).

Pod elongation after the pod lag phase continued similarly for primary and lateral racemes of all photoperiod and shading treatments in line with that reported by Zheng *et al.* (2003), who found that short days anticipated pod elongation but pod elongation rate remained unchanged. These results observed at the individual pod level are in line with those observed at the plant or community level, where partitioning of assimilates to pods was delayed but afterwards continued at the same rate when photoperiod was extended (Nico *et al.*, 2015). As pod elongation began later, but the subsequent elongation rate was not affected by photoperiod extension in our experiments, it seems that the photoperiodic response is associated with the onset of an ‘elongation signal’ rather than other attributes related to pod growth and development. This suggestion is reinforced by the microscopic observations of embryos developing under different photoperiods, which revealed that the internal embryo development (requiring low assimilate supply) was linked to the external pod’s development (which requires active biomass accumulation in the pod walls) irrespective of the pod’s chronological age.

Assuming that photoperiod is triggering a developmental elongation signal, the beginning of dry matter accumulation into pods and seeds could be uncoupled from the beginning of flowering, as suggested by Thomas and Raper (1976). This uncoupling could reproduce the effects observed on pod set when photoperiod is extended from flowering onwards and could be used as a favourable trait in soybean breeding programmes. Additionally, the ‘flowering and elongation signals’ should be independently regulated by photoperiod to mimic our treatments (initiated at R1). Some evidence of this independence has been found in other species. In groundnuts, photoperiod regulates the onset of pod growth but not flowering [*Arachis hypogaea* in Flohr *et al.* (1990); *Vigna subterranea* in Brink (1997)]. In potato, flowering and tuberization are photoperiodically regulated by two members of the potato FT-like gene family that respond to different environmental cues (Navarro *et al.*, 2011), suggesting that flowering and pod growth in soybean could also be partly independently regulated by photoperiod.

In conclusion, our results suggest that long days during post-flowering enhance pod number per node alleviating the competition between pods of different hierarchy. The photoperiodic effect on dominant pod development, delaying their elongation and therefore postponing their active growth, extends flowering and allows pod set at usually dominated positions. Some questions are still unanswered in relation to the nature of the interaction between dominant and dominated pods: Are long days altering the competition for assimilates between dominant and dominated pods? Or are they removing some sort of chemical inhibition? This is the subject of future research.

## Supplementary Data

Supplementary data is available at *JXB* online.

Supplementary Fig. S1. The dynamics of pod development presented in Fig. 2 at other node positions or under shading.

Supplementary Fig. S2. The relationship between the duration of the pod lag phase and pod number-determining variables.

Supplementary Fig. S3. The relationship between pod lag phase duration and the photoperiod explored during the day the flower opened.

Supplementary Data

## Abbreviations:

A: open flower
BF: pod with seeds >3mm long
P_0_: pod <1cm long
P_1_: pod 1–2cm long
P_2_: pod 2–3cm long
P_3_: pod 3–4cm long
P_4_: pod 4–5cm long
P_5_: pod >5cm long
PPN: pods per node
td: thermal days.
