# Supplementary material for: Soybean fruit development and set at the node level under combined photoperiod and radiation conditions
Source: J Exp Bot. 2015 Oct 27;67(1):365–77. doi: 10.1093/jxb/erv475 (PMC4682441; doi:10.1093/jxb/erv475)
Supplement: Supplementary Data [file supp_67_1_365__index.html]

Soybean fruit development and set at the node level under combined photoperiod and radiation conditions — Soybean fruit development and set at the node level under combined photoperiod and radiation conditions — Supplementary Data 

# Soybean fruit development and set at the node level under combined photoperiod and radiation conditions

## Supplementary Data

Data files

- Supplementary Data - Supplementary Data
