# Supplementary material for: Soybean fruit development and set at the node level under combined photoperiod and radiation conditions
Source: J Exp Bot. 2015 Oct 27;67(1):365–77. doi: 10.1093/jxb/erv475 (PMC4682441; doi:10.1093/jxb/erv475)
Supplement: Supplementary Data [file supp_erv475_supplementary_figures_S1_S3.pdf]

Journal of Experimental Botany

Supplementary Data

Title: Soybean fruit development and set at the node level under combined photoperiod and radiation conditions.

Authors: Nico, Magalí[1]*; Mantese, Anita I.[2]; Miralles, Daniel J.[3, 4, 5]; Kantolic, Adriana G.[1]

Figure S1. Developmental stage of the first fruit on the raceme as a function of thermal days after flowering (R1) for primary (full symbols and line) and lateral racemes (empty symbols and dotted line) at basal, central and apical nodes of mainstems of unshaded and shaded plants under control (circles), 1.5 (triangles) or 3 h extended photoperiod (squares), in Exp 1 and Exp2. Developmental scale: open flower (A), pod <1 cm long (P_0), pod 1 to 2 cm long (P_1) or pod >2 cm long (P_2) in both experiments. In Exp2 four extra categories were included: pod 3 to 4 cm long (P_3), pod 4 to 5 cm long (P_4), pod >5 cm long (P_5) and pod with seeds >3 mm long (BF). The panel of central nodes of unshaded plants in Exp 2 is also present on Figure 2 of the manuscript.

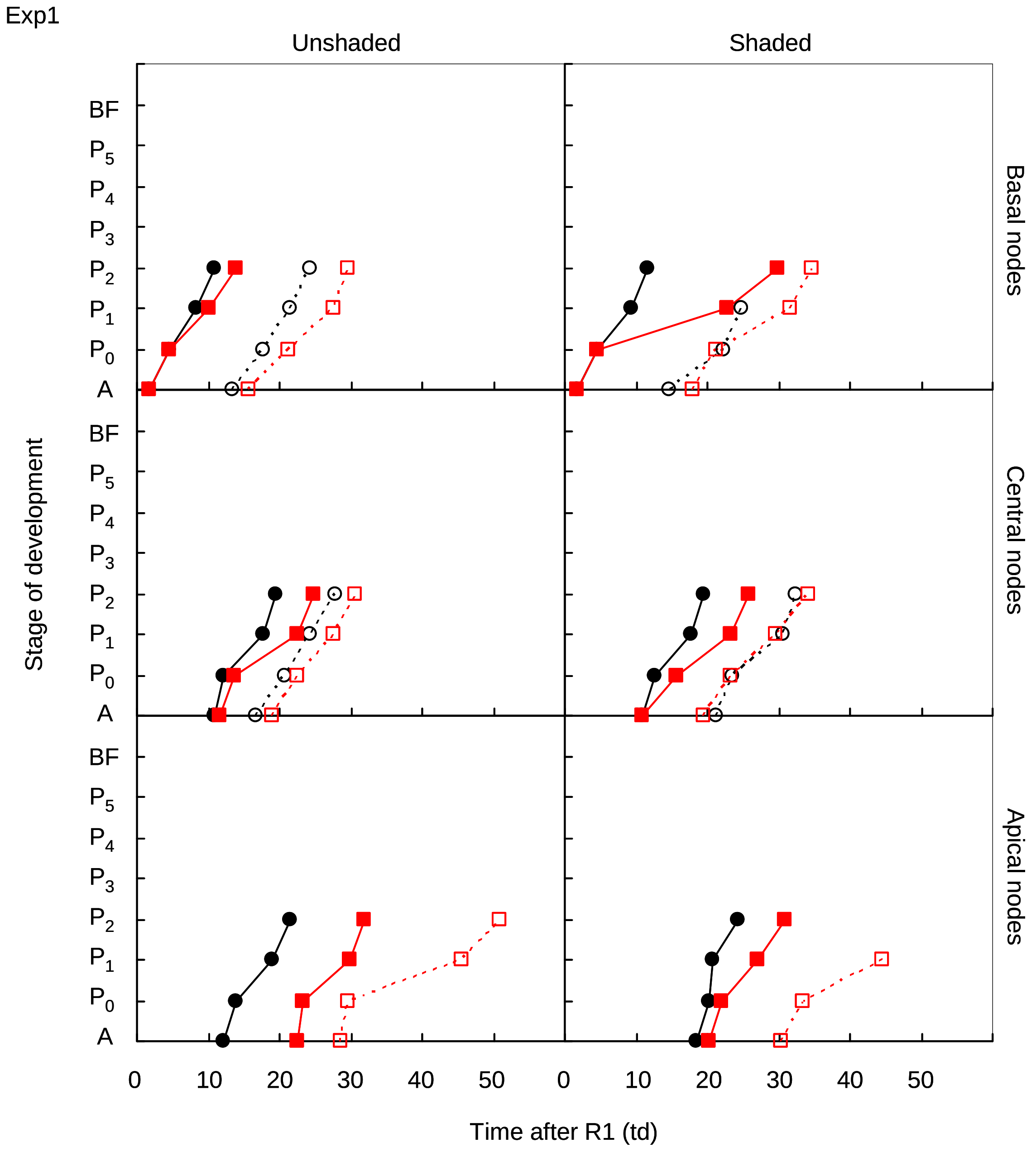

Exp2

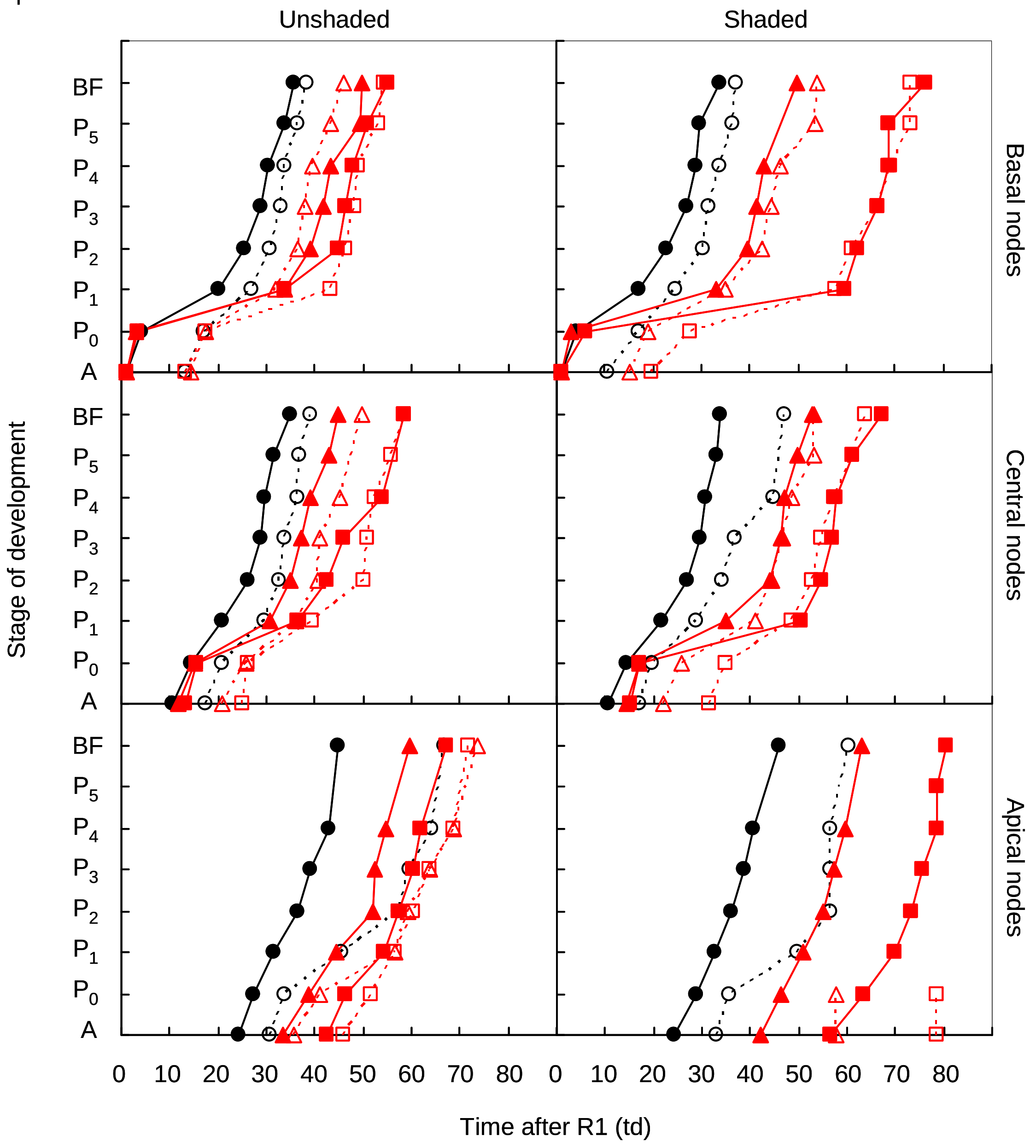

Unshaded
Shaded
BF
P_5
P_4
P_3
P_2
P_1
P_0
A
Stage of development
Basal nodes
Central nodes
Apical nodes
0 10 20 30 40 50 60 70 80
Time after R1 (td)

Figure S2. Mean number of flowers, pod set and number of pods in primary and lateral racemes as a function of pod lag phase (in thermal days) at basal (squares), central (circles) and apical nodes (triangles). The line and equation represent the fitted model for all node positions.

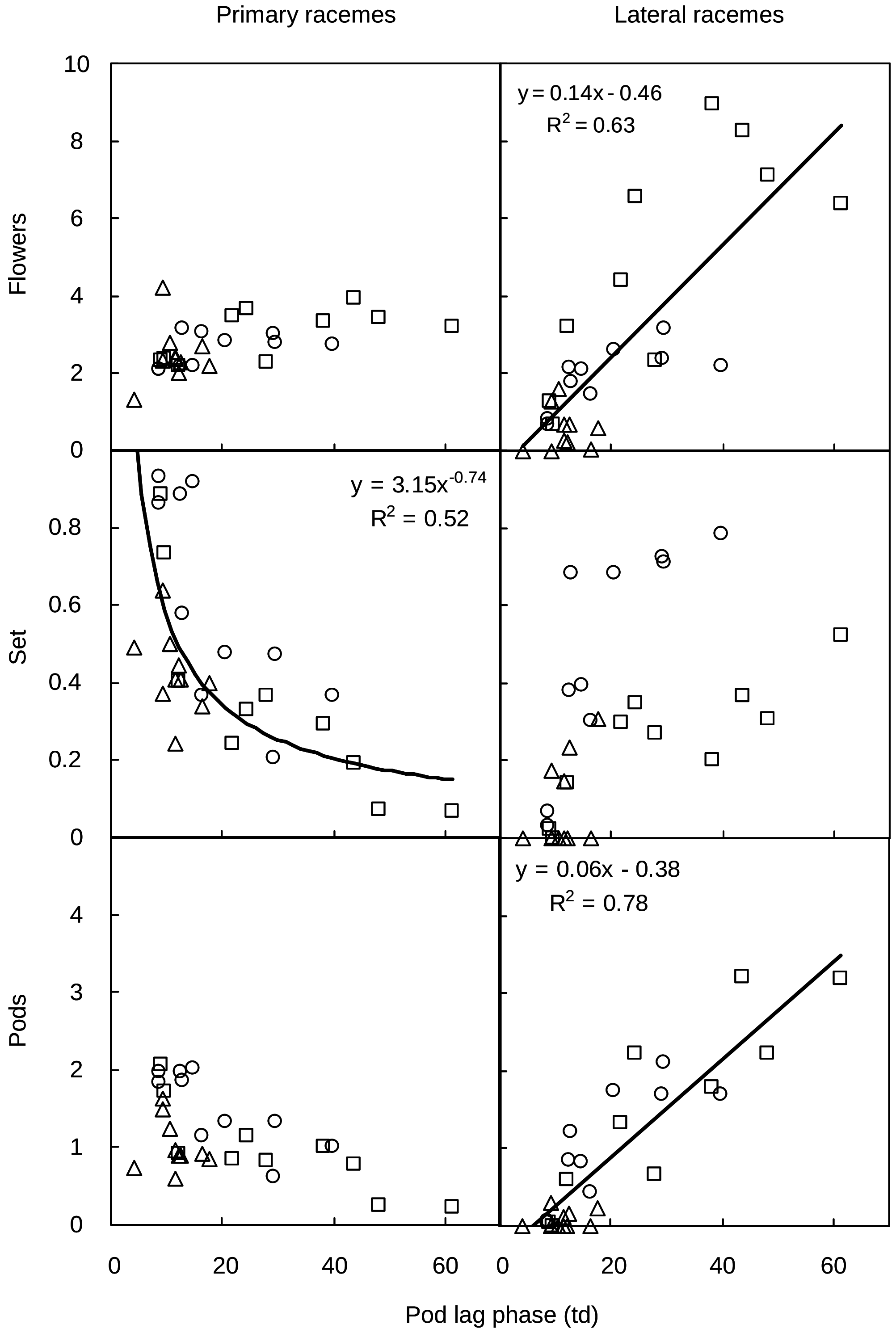

Figure S3. Pod lag phase (in thermal days) vs. mean photoperiod explored during the day the flower opened, for the first flower/pod of primary (full symbols and line) and lateral racemes (empty symbols and dotted line) at basal, central and apical nodes on the main stem. Equation variables are presented in the table below.

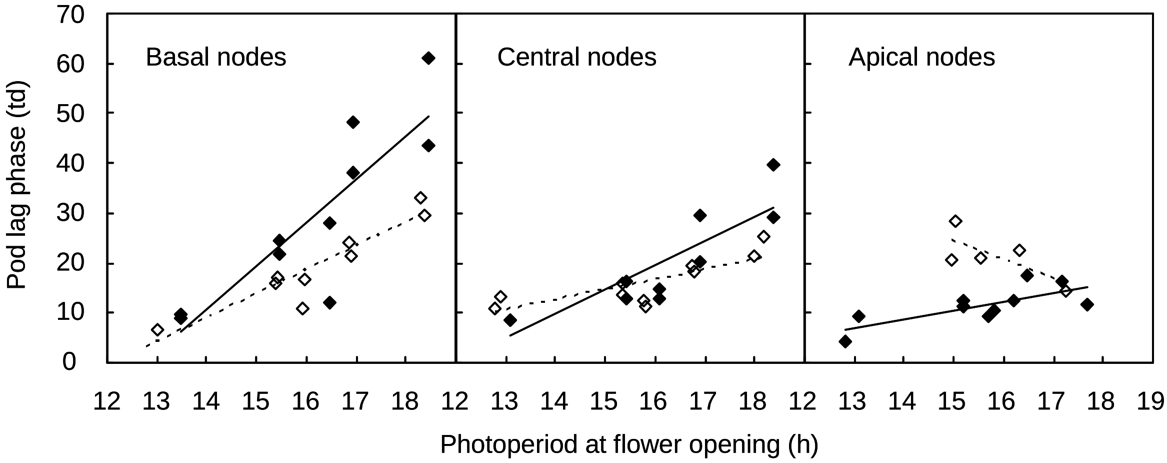

	Slope	Origin	r^2	p-value
**Basal nodes**				
Primary racemes	8.68	-110.6	0.73	0.0013
Lateral racemes	4.62	-56.27	0.85	0.0004
**Central nodes**				
Primary racemes	8.20	-57.61	0.74	0.0015
Lateral racemes	2.05	-16.24	0.64	0.0054
**Apical nodes**				
Primary racemes	1.83	-17.06	0.56	0.0124
Lateral racemes	-4.03	84.98	0.62	0.1137
